# Primary, cardiac, fibroblastic osteosarcoma: A case report

**DOI:** 10.1097/MD.0000000000009543

**Published:** 2018-01-05

**Authors:** Yangyang Li, Teng Ye, Qianru Gu, Lei Dong, Guorong Chen, Shanshan Lu

**Affiliations:** a Department of Pathology, The First Affiliated Hospital of Wenzhou Medical University; b Department of Dermatology, Wenzhou Hospital of Integrated Chinese and Western Medicine, Wenzhou Children's Hospital, Wenzhou, Zhejiang; c Department of Pathology, Rui Jin Hospital, Shanghai Jiao Tong University School of Medicine, Shanghai, China.

**Keywords:** fibroblastic, osseous differentiation, pathological features, primary cardiac fibroblastic osteosarcoma

## Abstract

**Rationale:**

Primary cardiac osteosarcoma is a rare tumor. To our knowledge, only 15 cases have been reported in the literature in the past 10 years. We describe a case of primary, cardiac, fibroblastic osteosarcoma in a 42-year-old woman.

**Patient concerns:**

A 42-year-old woman with a 10-day history of chest pain. Intraoperatively, a mass was found originating from the ostium of the left inferior pulmonary vein in the left atrium, extending to the mitral orifice. Histologically, the tumor contained variable amounts of spindle cells and osseous differentiation in different areas. Primary, cardiac fibroblastic osteosarcoma had the typical appearance of interlacing hyperchromatic spindle-shaped stromal cells associated with osseous matrix.

**Diagnoses:**

According to the clinicopathological features, diagnosis of primary, cardiac fibroblastic osteosarcoma was made.

**Interventions:**

Wide surgical excision of the mass was performed.

**Outcomes:**

Three months after the operation, transthoracic echocardiography demonstrated a 3.2 cm × 2 cm recurrent mass in the wall of the left atrium (LA). She died shortly afterwards as a result of the local disease recurrence.

**Lessons:**

In this report, we describe a rare case of primary, cardiac fibroblastic osteosarcoma, and findings are helpful for the pathologists would like to further identify the clinicopathological features of this rare tumor.

## 1 Introduction

Primary cardiac osteosarcoma was a rare, intracardiac tumor. Only a few reports of cardiac osteosarcoma had been published in the literature. Due to the rarity of this subtype, its clinicopathological feature is poorly understood. Hence, we described a case of primary, cardiac, fibroblastic osteosarcoma. The patient gave consent for these studies and their publication.^[1,2]^

## 2 Case presentation

A 42-year-old woman, presented with a history of hemoptysis for 1 month. She attended the First Affiliated Hospital of Wenzhou Medical University with a 10-day history of chest pain. There was no history of malignant thromboembolic, hematologic, or inflammatory disease. Physical examination showed her blood pressure was 126/79 mmHg, her pulse was regular with a rate of 72/min, and her breathing rate was 20/min. A systolic rumble at the apex area was noted. Arterial blood gas analysis showed pH of 7.39, *P*O_2_ of 176.7 mmHg, *P*CO_2_ of 39.1 mmHg, and oxygen saturation of 99.5% under a reserved O_2_ mask. Transthoracic echocardiography demonstrated a large, immobile, ovoid mass attached to the wall of the left atrium (LA); the mitral valve was involved. Echocardiography revealed moderate, mitral insufficiency, and an abnormal echoic mass in the left atrium. A diagnosis of primary cardiac tumor without metastases was considered in the clinic. Intraoperatively, a mass was found originating from the ostium of the left inferior pulmonary vein in the left atrium, extending to the mitral orifice. Wide surgical excision of the mass was performed. Macroscopically, the specimen measured 5.5 cm × 5 cm × 4 cm. The tumor was grey, solid, and firm.

**Figure 1:**
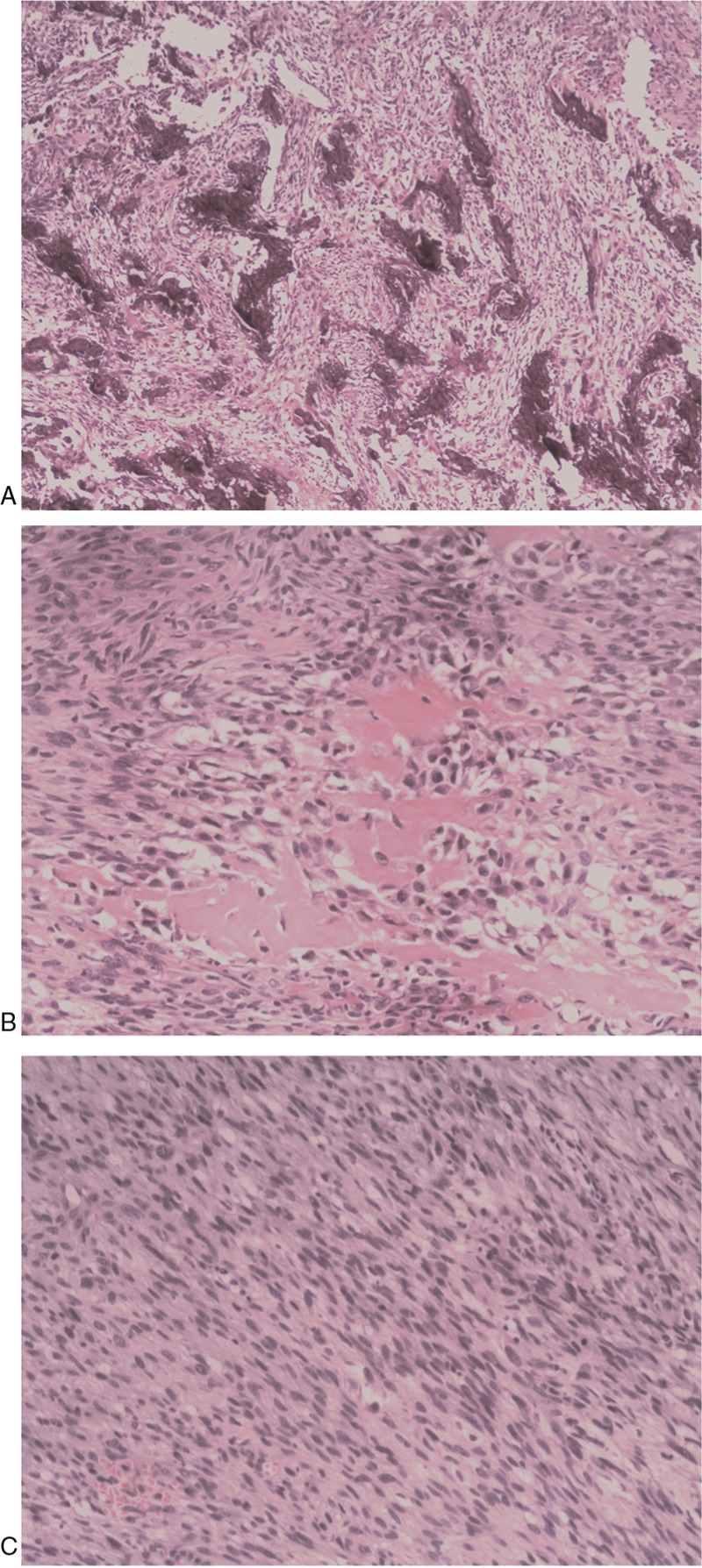
A: Primary cardiac fibroblastic osteosarcoma with well-differentiated osteosarcoma infiltrating the left atrium (HE, ×4 magnification). B: Primary cardiac fibroblastic osteosarcoma with osseous matrix, osteoid matrix embedded pleomorphic tumor cells (HE, ×20 magnification). C: Spindle cells with a solid growth pattern of pleomorphic and spindle cells with prominent nuclear atypia (HE, ×10 magnification).

Histologically, the tumor contained variable amounts of spindle cells and osseous differentiation in different areas. Primary, cardiac fibroblastic osteosarcoma had the typical appearance of interlacing hyperchromatic spindle-shaped stromal cells associated with osseous matrix (Fig. 1A). Osteoclastic-like, giant cells were scattered throughout without foci of hemorrhage and necrosis (Fig. 1B). Tumor cells were pleomorphic with spindle-shaped nuclei and hyperchromatin (Fig. 1C). Immunohistochemical examination showed that tumor cells were positive for vimentin, S-100 protein, and Bcl-2, but negative for cytokeratin, epithelial membrane antigen (EMA), smooth muscle actin, CD99, and CD34. The proliferative index (Ki-67) was approximately 60%. The genetic profile of this tumor by fluorescence in situ hybridization (FISH) showed the amplification of mdm-2 genes. These findings were compatible with a histological diagnosis of fibroblastic osteosarcoma. She was asymptomatic in the immediate postoperative phase. However, 3 months after the operation, her condition worsened and transthoracic echocardiography demonstrated a 3.2 cm × 2 cm recurrent mass in the wall of the left atrium (LA). She died shortly afterwards as a result of the local disease recurrence.

## 3 Discussion

Primary cardiac osteosarcoma is a rare, intracardiac tumor. The name was first coined by Cumming and Shillitoe^[3]^ in 1957, since then, only a few reports of cardiac osteosarcoma have been published in the literature, most of them were isolated cases or small series.^[1]^ The age of patients at diagnosis ranges from 14 to 77 years old, the median age is in the 4th decade. Women are affected more commonly than men.^[4]^ These cardiac tumors present with a variety of symptoms, including those related to obstruction, embolism, and elaboration of substances resulting in constitutional symptoms. Their presentation depends not only upon the size of the tumor, but also upon its anatomic location. Growth rate, friability, and invasiveness are also important factors that determine clinical features.^[5,6]^ The optimal approach to treatment of primary cardiac fibroblastic osteosarcoma remains unknown. Complete surgical excision is recommended.

Virtually all osteosarcomas of the heart arise in the left atrium,^[4,7,8]^ By contrast, osteosarcomas metastatic to the heart most commonly involve the right side of the heart.^[4]^ Tumors in the left atrium can obstruct the mitral or aortic valve. If the tumor obstructs the pulmonary venous return to the left side of the heart, it may lead to pulmonary congestion and edema. Symptoms such as dyspnea and orthopnea are common in these circumstances.^[6]^ Persistent cough is the most common symptom, but other clinical manifestations such as chest pain and hemoptysis may also be present. At gross examination, primary cardiac osteosarcoma may appear soft and granular (osteolytic) or sclerotic and dense (osteosclerotic), depending on the degree of mineralization. Adjacent soft tissue extension is frequent.^[9]^ At histologic examination, osteosarcomas can be subgrouped into osteoblastic and fibroblastic types, depending on the predominant component.^[10]^ The osteosarcomas are classified as fibroblastic variant when tumor cells are spindle-shaped and arranged in interlacing fascicles.^[11]^ The osteoid tissue (a precursor of bone) is present within a sarcomatous stroma. The stromal cells may show anaplasia; their shape varies from spindles to round, and the cells contain hyperchromatic nuclei. The degree of vascularization varies considerably from scant to abundant.^[9]^ Unlike primary, cardiac, fibroblastic osteosarcoma, myxoma is largely made up of amorphous, mucopolysaccharide-rich matrix conferring the typically “myxoid” aspect whereas fibrosarcoma is composed of mono-morphic spindle cells, with variable mitotic activity arranged in fascicles.

This case is a biphasic tumor composed of two cell types; spindle-shaped and epithelioid cells. It is positive for cytokeratin and epithelial membrane antigen (EMA). There is no osseous differentiation. In contrast with primary, cardiac, fibroblastic osteosarcoma, metastatic carcinoma demonstrates cellular pleomorphism from any extra-cardiac malignant neoplasm able to spread to distant sites. Osteoid is eosinophilic with hematoxylin-eosin staining and may resemble collagen when it presents in small quantities; immunohistochemical stains can help in differentiating the 2. On the basis of the predominant component of the stroma, lesions can be subtyped as osteoblastic or fibroblastic. These tumors are uniformly positive for vimentin. Although the best approach to the treatment of primary cardiac fibroblastic osteosarcoma remains unknown, complete surgical excision is recommended. Radiation therapy and chemotherapy are not very effective.^[12]^ Primary, malignant cardiac tumors, including osteosarcoma, are extremely rare and continue to be associated with a poor prognosis.^[13]^ Survival time varies greatly; 5 cases in previous reports died within 1 month of the operation, while the longest survival time was 131 months postoperatively.^[14]^
